# Nutritional composition of “gari” analog produced from cassava (*Manihot esculenta*) and cocoyam (*Colocasia esculenta*) tuber

**DOI:** 10.1002/fsn3.165

**Published:** 2014-09-02

**Authors:** Oluwaseun P Bamidele, Femi G Ogundele, Basirat A Ojubanire, Mofoluwaso B Fasogbon, Olayide W Bello

**Affiliations:** 1 Department of Food Science and Technology, Obafemi Awolowo UniversityIle-Ife, Osun State, Nigeria; 2 Department of Food Science and Technology, Federal Polytechnic OffaOffa, Kwara State, Nigeria

**Keywords:** Antinutrient and minerals, cassava tuber, cocoyam tuber, proximate composition

## Abstract

Physicochemical properties of*gari* analog produced from coprocessing of Cassava (*Manihot esculenta Crantz*) and Cocoyam (*Colocasia esculenta*) were investigated. Cassava tuber and cocoyam were coprocessed at different percentages before frying separately. Proximate composition, mineral content, antinutritional factors, and sensory evaluation of various samples were determined. The results showed that the moisture content of all the samples was in the same range (7.28 ± 0.30 to 7.78 ± 0.14%). The protein content (1.57 ± 0.14 to 4.43 ± 0.16), ash (1.89 ± 0.10 to 2.15 ± 0.30), and crude fiber (1.53 ± 0.50 to 2.19 ± 0.10%) showed a significant increase with increase in the level of cocoyam substitution. The fat and carbohydrate content decreased with an increase in cocoyam level. The mineral contents of the samples increased with an increase in cocoyam content with sample F having the highest value of potassium, followed by samples E and D (68 mg/100 g, 35 mg/100 g, and 24 mg/100 g). The antinutritional factors of all the samples were at very low concentration while samples B, C, and D competed favorably with sample A (control) in sensory evaluation. In conclusion, coprocessing of cassava and cocoyam improved the nutritional quality of the*gari* produced with high level of acceptance from the taste panelist.

## Introduction

Cassava (*Manihot esculenta Crantz*) is to African villager farmers as rice is to the Asian farmer, or potato and wheat are to European farmers (Montagnac et al. 2009). It has so many names aside from cassava, such as manioc, mandoica, and it is the most important food in terms of carbohydrates (Ojo and Akande 2013). Cassava is eaten daily in various forms such as*gari*, fufu, and tapioca (Okechukwu and Okoye 2010).*Gari* is a lactic acid–fermented product of cassava root that can be processed with palm oil rich in carotenoid (“yellow gar”) or without palm oil. In Nigeria,*gari* is widely acceptable and consumed by both the poor, the middle men or average Nigerian, and also the rich because it serves as a major source of carbohydrate.*Gari* can be taken in various forms; some people use it to make*eba* or soak inside water along with groundnut, mashed beans, or bean cake (*akara*). The major problem of consuming*gari* is the toxicity which may arise from poor processing of cassava which is rich in cyanogenic glucosides. Consumption of cyanide and its accumulation in human body normally lead to neurological disorders and goiter (Ojo and Akande 2013). Cyanide has been found to be greatly reduced during the processing of cassava to*gari*. Unit operation such as peeling, washing, grating, fermentation, dewatering, and roasting have been found to effectively reduce the residual cyanide contents of the product (Ojo and Akande 2013). Chijioke et al. (2010) reported that the traditional method of*gari* production which requires the cassava slurry to be fermented for 72 h during which the cyanides (linamarin and lotaustralin) are hydrolyzed by linamarase enzyme to yield hydrocyanic acid which has low boiling point and easily escape during roasting render the*gari* safe for consumption. Cutting corners by so many processors for the sake of profit has led to production of*gari* with excess cyanide content (Ojo and Akande 2013). Cocoyam (*Colocasia esculenta*) belongs to Araceae family and constitutes one of the six most important roots and tuber crops worldwide (Ndabikunze et al. 2011). Nigeria, Ghana, Burundi, Cote d'Ivoire and Madagascar, China, Japan, Philippines, and Thailand account for the production of 8.3 million tons of cocoyam per year (FAO 1998). Cocoyam is mainly cultivated by small-scale farmers in most African countries (Ndabikunze et al. 2011). Like other members of Araceae family, it grows from the fleshy tuber, which is used majorly for food, and it supplies digestible starch, substantial amount of protein, vitamin C, riboflavin, thiamine, niacin, and significant amount of dietary fiber that is richer than cassava tuber (Niba 2003). The production of cocoyam is low when compared with other roots and tuber in Nigeria (Aderolu et al. 2009). In spite of its importance as a staple food in many countries, cocoyam has received very poor research attention to enhance its utility and production (Watanabe 2002). Despite the nutritional composition of digestible starch of 98.8% and its richness in sulfur amino acid, the potential for the development of value-added cocoyam products has not been exploited (Palapala et al. 2005; Ndabikunze et al. 2011). Promotion and supporting the use of cocoyam can make a major contribution to the food security of the countries where cocoyam is being cultivated. Hence, this study aimed at producing “gari” using cassava and cocoyam tubers to increase the utilization of cocoyam and to analyze the physicochemical properties of “gari.”

## Materials and Methods

**Figure 1:**
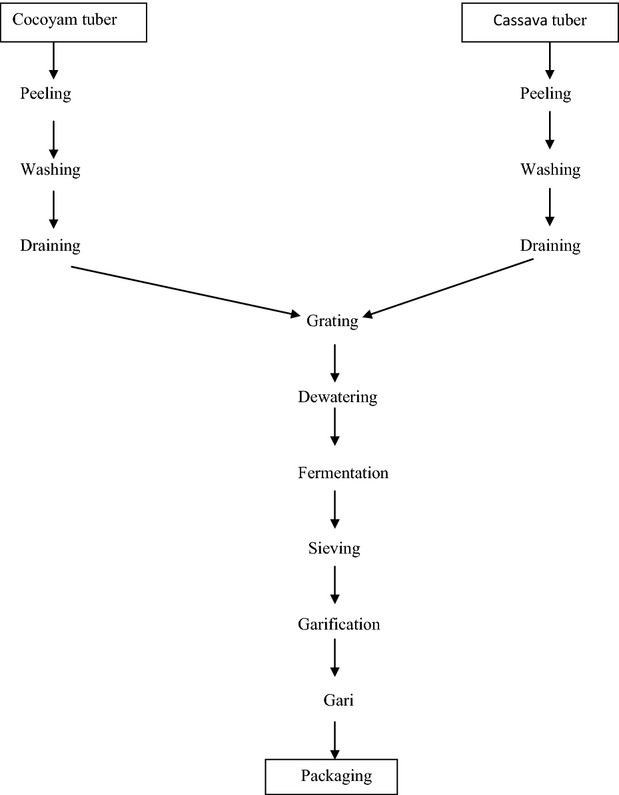
Processing of “gari” from cassava and cocoyam tubers.

Cassava and Cocoyam tubers were collected from Obafemi Awolowo University Teaching and Research farm in Ile-Ife, Osun State, Nigeria. The tubers were processed separately (i.e., sorted, washed, peeled, and diced). The diced tubers were cograted at different ratios of cassava to cocoyam tuber. Sample A serves as the control which was 100% cassava, 80% of cassava tuber to 20% of cocoyam serves as sample B, 70% of cassava tuber to 30% of cocoyam serves as sample C, 50% cassava to 50% cocoyam serves as sample D, and sample E comprises 40% cassava to 60% cocoyam, while sample F was 100% cocoyam. After cograting, the samples (A–F) were*gari*fied separately by roasting in a deep frying pan. The “gari” produced were cooled in a tray and packed inside polyethylene bags for analyses. Figure1 shows the step-by-step processing procedure for “gari” production.

### Proximate composition

Proximate composition of the “gari” samples for moisture, ash, fat, and protein contents were determined using Association of Official Analytical Chemists (2005) methods. Total carbohydrate content was determined by subtracting the ash, protein, and fat percentages from 100%.

### Functional property determination

#### Water absorption determination

Water absorption was determined by the modified centrifuge method of Lin and Zayas (1987). Each sample (2.0 g) was transferred into a lagged 50 mL centrifuge tube and weighed (W_1_). While 30 mL of hot distilled water at 70°C was added to each sample, simultaneously washing down the inside of the centrifuge tubes using a glass stirring rod, the sample and the water was mixed for 30 min. The suspension was allowed to rest for 10 min and centrifuged at 1165*g* for 25 min at 50°C. The tube was cooled in a desiccator and weighed (W_2_).


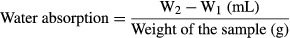


#### Swelling capacity determination

Swelling capacity was determined by modification of the Lin and Zayas (1987) method. Each sample (2 g) was dispersed in 40 mL distilled water. The resultant slurry was heated at a temperature of 70°C for 30 min in a water bath, cooled to room temperature, and centrifuged at 598*g* for 30 min. The supernatant liquid was decanted and the centrifuge tube was dried for 25 min at 50°C inside a hot air oven. The residue was weighed (W_2_). The centrifuge tube containing the sample alone was weighed prior to adding distilled water (W_1_).

#### Bulk density determination

Bulk density was determined using the gravimetric method as described by Okaka and Potter (1979). Each sample (10 g) was weighed into a 25 mL graduated cylinder. The cylinder was gently tapped 10 times against the palm of the hand. The bulk density was expressed as the sample per volume occupied by the sample.

#### Mineral analysis

The mineral analysis was determined by the method described by AOAC (2005). The samples were ashed at 550°C. The ash obtained was boiled with 10 mL of 20% hydrochloric acid in a beaker and then filtered into a 100 mL standard flask. The filtrate was made up to the mark with de-ionized water. The minerals sodium (Na) and potassium (K) were determined from the solution using the standard flame emission photometer. NaCl and KCl were used as the standards (AOAC 2005). Phosphorus was determined calorimetrically using the spectronic 20 (Gallenkamp, UK; Kirk and Sawyer 1991) with KH_2_PO_4_ as the standard. Calcium (Ca), magnesium (Mg), and iron (Fe) were determined using an atomic absorption spectrophotometer (AAS, Model SP9, Pye Unicam Ltd, Cambridge, UK). All values were expressed in mg/100 g.

### Antinutritional factor determination

The antinutrients saponin, calcium oxalate, trypsin inhibitors, tannins, and phytate levels in the samples of “gari” produced were determined using the rapid test method of AOAC (2005).

#### Sensory evaluation of the “gari”

The*gari* samples produced were subjected to a sensory test using 10 panelists. The products were rated in terms of taste, color, texture, aroma, and overall acceptability on a 9-point hedonic scale ranging from 9 (dislike extremely) to 1 (like extremely) and the results generated were analyzed using analysis of variance (ANOVA).

### Statistical analysis

The data were analyzed using SPSS version 20.0 (IBM SPSS Statistics for Windows, IBM Corp., Armonk, NY). The mean and standard deviation (SD) of the triplicate analyses were calculated. ANOVA was performed to determine significant differences between the means, while the means were separated using the new Duncan multiple range test.

## Results and Discussions

### Proximate composition

The proximate compositions of the*gari* produced are shown in Table1. The moisture content of all the samples ranged between 7.28% and 7.78%, with sample A having the least value. The moisture content of all the samples was in the range recommended by Abu et al. (2006) which indicated that the maximum moisture content of*gari* is 12%. This result is consistent with the report of Ojo and Akande (2013). The protein content, ash, and crude fiber of all the samples were higher than that of sample A which serves as the control. The protein content of sample A was 1.57 ± 0.14% and it was the least value. Protein content of the other samples increased with an increase in the percentage of cocoyam substituted into cassava in producing the*gari*. This may be due to the high protein content of cocoyam as reported by Ndabikunze et al. (2011). Increase in protein content of the*gari* was as a result of availability of total essential amino acids and higher protein digestibility in the samples (Ihekeronye and Ngoddy 1985; Ojo and Akande 2013). The ash content ranged from 1.89 ± 0.10% for sample A to 2.15 ± 0.30% for sample F. Increase in ash content up to 0.5% was an indication of an increase in the mineral content of the product (Adeleke and Odedeji 2010). The crude fiber content also ranged between 1.53 ± 0.50% and 2.19 ± 0.10%. The increase in crude fiber of all the samples may be due to higher crude fiber of cocoyam that was added to that of cassava at different percentages. This result is consistent with the report of Ojo and Akande (2013) who reported an increase in crude fiber content of*gari* produced from cassava and potato. The fat contents of the samples decreased with an increase in percentage of cocoyam. This may be due to cocoyam having low content of fat as reported by Ndabikunze et al. (2011) and Lewu et al. (2010). The carbohydrate content showed that sample A had the highest value of 86.25 ± 0.17% followed by sample B (85.65 ± 0.12%). However, all the samples had a good quantity of carbohydrate with the addition of cocoyam.

**Table 1:** Proximate composition of*gari* analog produced from cassava (*Manihot esculenta Crantz*) and cocoyam (*Colocasia esculenta*) tubers (%).

Samples	Moisture	Protein	Crude fat	Ash	Crude fiber	Carbohydrate
A	7.28 ± 0.30^b^	1.57 ± 0.14^a^	1.48 ± 0.20^c^	1.89 ± 0.10^a^	1.53 ± 0.50^a^	86.25 ± 0.17^e^
B	7.29 ± 0.25^a^	2.23 ± 0.10^b^	1.25 ± 0.10^a^	2.07 ± 0.12^b^	1.87 ± 0.34^b^	85.65 ± 0.12^d^
C	7.53 ± 0.42^c^	3.50 ± 0.03^c^	1.27 ± 0.21^b^	2.02 ± 0.17^b^	1.89 ± 0.41^b^	82.79 ± 0.35^b^
D	7.66 ± 0.10^d^	3.58 ± 0.22^c^	1.26 ± 0.33^b^	2.18 ± 0.41^c^	2.02 ± 0.16^c^	83.27 ± 0.22^c^
E	7.74 ± 0.16^e^	3.96 ± 0.22^d^	1.21 ± 0.10^b^	2.15 ± 0.11^c^	2.17 ± 0.12^d^	82.77 ± 0.15^b^
F	7.78 ± 0.14^e^	4.43 ± 0.16^e^	1.22 ± 0.35^b^	2.15 ± 0.30^c^	2.19 ± 0.10^d^	82.27 ± 0.15^a^

Values are means and standard deviation of triplicate of three samples (*n* = 9); means followed by the same letter within the same row are not significantly different (*P* > 0.05).

### Functional properties

Table2 shows the functional properties of the*gari* analog produced from cassava and cocoyam. Functional properties of any sample are an indication of usefulness of such food samples for various food products. The water absorption capacity of sample A was the highest (150.46 g/mL) followed by sample B (130.21 g/mL), then samples C, D, E, and F (130.00, 120.84, 120.22, and 100.26 g/mL), respectively. This result revealed a decrease in the water absorption capacity of the samples followed up by the addition of cocoyam to cassava. This may be due to the low retention power of cocoyam to entrap large amounts of water. Chen and Lin (2002) reported that the water absorption capacity of any food product, either flour or grain, is the ability of such product to entrap a large amount of water. This result was supported by Ezeocha et al. (2011) who reported a low water absorption capacity of cocoyam composite flour for fufu production. The swelling capacity also followed the same trend exhibited by water absorption capacity. The highest value was obtained for sample A followed by samples B, C, D, E, and F (220.24, 201.15, 194.51, 190.81, 190.42, and 174.26 g/ml, respectively). The entrapped water by the food molecule will be useful in making the food sample to swell (Ezeocha et al. 2011). The bulk density results can be grouped into two categories, with samples A and B making one category (8.21 and 8.04 g/mL), and samples C, D, E, and F making the second (7.66, 7.64, 7.55, and 7.54 g/mL), respectively. The bulk density is always influenced by particle size and the density of such food product which determines the packaging and handling method of such material (Karuna et al. 1996; Ezeocha et al. 2011).

**Table 2:** Functional properties of*gari* analog produced from cassava (*Manihot esculenta Crantz*) and cocoyam (*Colocasia esculenta*) tubers (g/mL).

Samples	Water absorption capacity	Swelling capacity	Bulk density
A	150.46 ± 0.31^d^	220.24 ± 0.11^e^	8.21 ± 0.10^d^
B	130.21 ± 0.50^c^	201.15 ± 0.35^d^	8.04 ± 0.12^c^
C	130.00 ± 0.20^c^	194.51 ± 0.21^c^	7.66 ± 0.30^b^
D	120.84 ± 0.11^b^	190.81 ± 0.15^b^	7.64 ± 0.12^b^
E	120.22 ± 0.15^b^	190.42 ± 0.22^b^	7.55 ± 0.13^a^
F	100.26 ± 0.14^a^	174.26 ± 0.10^a^	7.54 ± 0.15^a^

Values are means and standard deviation of triplicate of three samples (*n* = 9); means followed by the same letter within the same row are not significantly different (*P* > 0.05).

### Mineral composition

The mean values of the mineral composition of*gari* analog produced from cassava and cocoyam are shown in Table3. Calcium, sodium, potassium, magnesium, iron, and phosphorous were tested. For all the samples, sample A which serves as the control (100% cassava*gari*) exhibited the least value of all the minerals. Sample F (100% cocoyam*gari*) exhibited the highest value. This may be due to the fact that cocoyam is richer in mineral composition than cassava. Potassium was the most abundant mineral with values ranging between 0.28 mg/100 g in sample A and 69.84 mg/100 g in sample F. Magnesium was the next abundant mineral with values ranging between 1.30 mg/100 g for sample A and 46.17 mg/100 g in sample F. The samples were very low in iron with sample F still leading with 7.89 mg/100 g and sample A with the least (0.17 mg/100 g). The value of phosphorus of all the samples was quite higher than that of calcium. The result is an indication of usefulness of cocoyam in increasing the mineral composition of food. Cocoyam can be considered a good source of potassium, magnesium, and sodium. This result is consistent with the findings of Lewu et al. (2010).

**Table 3:** Minerals composition of*gari* analog produced from cassava (*Manihot esculenta Crantz*) and cocoyam (*Colocasia esculenta*) tubers (mg/100 g).

Samples	Ca	Na	K	Mg	Fe	P
A	1.02	0.22	0.28	1.30	0.17	1.20
B	3.53	2.89	2.71	2.55	1.16	2.52
C	5.27	4.22	17.48	15.12	2.55	10.21
D	5.84	4.89	24.66	16.12	2.89	11.12
E	10.24	6.78	35.42	26.72	5.48	12.22
F	15.46	30.42	69.84	46.17	7.89	18.46

Values are means of triplicate of three samples (*n* = 9).

### Antinutritional factor

The results of the antinutritional content of*gari* analog produced from cassava and cocoyam are shown in Table4. The antinutritional factors determined were saponin, calcium oxalate, trypsin inhibitor, glycoside, and phytate. Sample A contained the largest amount of all the antinutritional factors tested – it ranged from 0.05 mg/100 g tannin to 0.01 mg/100 g phytate. For samples B and C, the entire antinutritional factor ranged from 0.01 mg/100 g saponin and 0.05 mg/100 g tannin, which was an indication that the samples contained very low antinutritional factor. The remaining samples (D, E, and F) followed the same pattern like others, with the value of antinutritional factors ranging between 0.00 mg/100 g saponin and 0.03 mg/100 g tannin. This may be due to the processing method used which required heat. Ojo and Akande (2013) reported that different processing methods such as cooking, autoclaving, and soaking have an influence in reducing the antinutritional factor of foods. The toxic compound hydrocyanic glycosides and other antinutrients were found below the permissible level (1%), indicating that the samples were safe for consumption.

**Table 4:** Antinutritional factor of*gari* analog produced from cassava (*Manihot esculenta Crantz*) and cocoyam (*Colocasia esculenta*) tubers (g/100 g).

Samples	Saponin	Calcium oxalate	Trypsin inhibitor	Glycoside	Tannin	Phytate
A	0.02	0.04	0.04	0.03	0.05	0.01
B	0.01	0.04	0.03	0.02	0.05	0.01
C	0.01	0.02	0.03	0.01	0.04	0.01
D	0.00	0.02	0.02	0.00	0.03	0.01
E	0.00	0.01	0.02	0.00	0.03	0.00
F	0.00	0.01	0.01	0.00	0.02	0.00

Values are means of triplicate of three samples (*n* = 9).

### Sensory evaluation

Table5 shows the mean sensory scores of all the*gari* samples made from the mixture of cassava and cocoyam. All the samples except samples E and F competed favorably with sample A. Samples A, B, C, and D were rated similarly with a slight difference in value. The values range from 7.04 in taste for sample D and 7.84 in texture for sample A. The remaining samples (E and F) were rated low (4.31 in flavor to 6.94 in texture). Sample E contained 60% of cocoyam and 40% of cassava; this may be the reason why it received a low rating due to change in taste, color, and texture of its*gari*. Sample F was 100% cocoyam*gari* and this may also be the reason for its low rating.

**Table 5:** Sensory evaluation result of the*gari* analog from cassava (*Manihot esculenta Crantz*) and cocoyam (*Colocasia esculenta*) tubers.

Samples	Taste	Color	Texture	Flavor	Overall acceptability
A	7.31	7.62	7.84	7.54	7.56
B	7.30	7.58	7.82	7.50	7.54
C	7.22	7.55	7.64	7.52	7.51
D	7.01	7.04	7.23	7.48	7.50
E	5.62	6.54	6.94	5.96	5.23
F	5.40	5.36	4.67	4.31	4.50

Values are means of triplicate of three samples (*n* = 9).

## Conclusion

The present study revealed the importance of cocoyam and one major aspect of its utilization in the food industry.*Gari* produced from 50% cassava and 50% cocoyam brought a significant increase to the nutritional composition of the product. Protein content (3.58%), fat content (1.26%), ash, crude fiber, and carbohydrate were all higher than the control (2.18%, 2.02%, and 83.27%). Also, the increase in the mineral content of the*gari* analog of sample D (50% cassava and 50% cocoyam) shows the usefulness of cocoyam as a supplement in*gari* production. Comilling of cassava and cocoyam helped in improving the nutritional and mineral qualities of the*gari*. It can be concluded that*gari* analog (*gari* from cassava and cocoyam) is better than*gari* prepared from cassava alone.

## Conflict of Interest

None declared.
